# Supplementary material for: Yeast translation elongation factor eEF3 promotes late stages of tRNA translocation
Source: EMBO J. 2021 Feb 8;40(6):e106449. doi: 10.15252/embj.2020106449 (PMC7957392; doi:10.15252/embj.2020106449)
Supplement: Supplementary file 1 — Appendix [file EMBJ-40-e106449-s001.pdf]

SUPPLEMENTAL INFORMATION

FOR

eEF3 promotes late stages of tRNA translocation including E-tRNA release from the ribosome

Namit Ranjan[1,#,*], Agnieszka Pochopien[2,3,#], Colin Chih-Chien Wu[4,#], Bertrand Beckert[3], Sandra Blanchet[1], Rachel Green[4,5,*], Marina V. Rodnina[1,*], Daniel N. Wilson[2,3,*]

[1] Department of Physical Biochemistry, Max Planck Institute for Biophysical Chemistry, Am Fassberg 11, 37077 Göttingen, Germany.

[2] Gene Center, Department for Biochemistry and Center for integrated Protein Science Munich (CiPSM), University of Munich, Feodor-Lynenstr. 25, 81377 Munich, Germany

[3] Institute for Biochemistry and Molecular Biology, University of Hamburg, Martin-Luther-King-Platz 6, 20146 Hamburg, Germany

[4] Department of Molecular Biology and Genetics, Johns Hopkins University School of Medicine, MD21205 Baltimore, United States.

[5] Howard Hughes Medical Institute, Johns Hopkins University School of Medicine, MD21205 Baltimore, United States.

[#] These authors contributed equally

*Correspondence: daniel.wilson@chemie.uni-hamburg.de, rodnina@mpibpc.mpg.de, ragreen@jhmi.edu, namit.ranjan@mpibpc.mpg.de

Table of contents

Appendix Figure S1 – page 2
Appendix Figure S2 – page 3
Appendix Figure S3 – page 4-5
Appendix Figure S4 – page 6
Appendix Figure S5 – page 7-8
Appendix Figure S6 – page 9
Appendix Table S1 – page 10
Supplemental References – page 11

SUPPLEMENTAL FIGURES

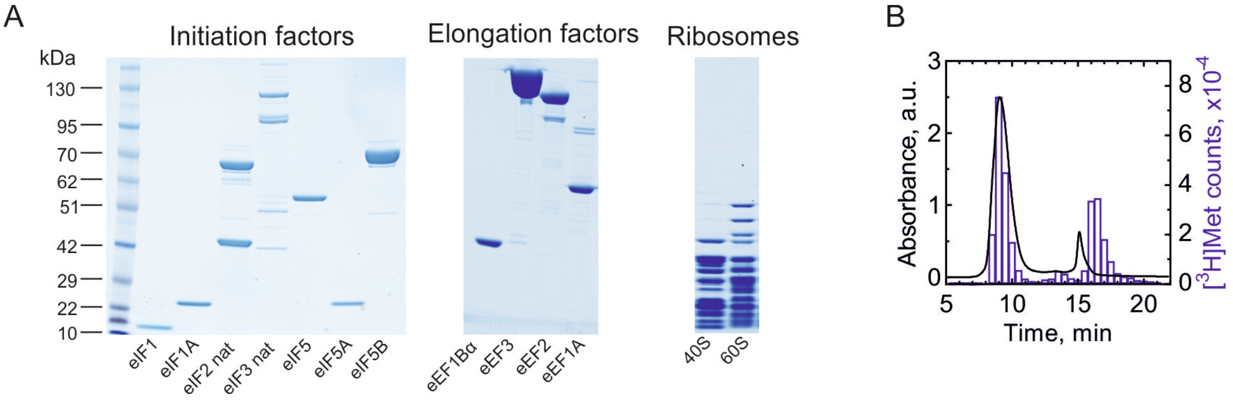

Appendix Figure S1 Components of the reconstituted yeast translation system, related to Figure 1. (**A**) Quality control of the initiation and elongation factors and the ribosomes by PAGE. For each component, 50 to 100 pmol were loaded on a NuPAGE 4 to 12% gradient gel. "nat" denotes purification of native eIF2 and eIF3 from yeast. (**B**) Purification of the 80S IC by SEC on a Biosuite 450 using HPLC. The presence of 80S IC is identified as the overlap between the absorbance of the ribosomes at 260 nm (black) and the radioactivity from [^{3}H]Met-tRNA$_{i}^{Met}$ (blue).

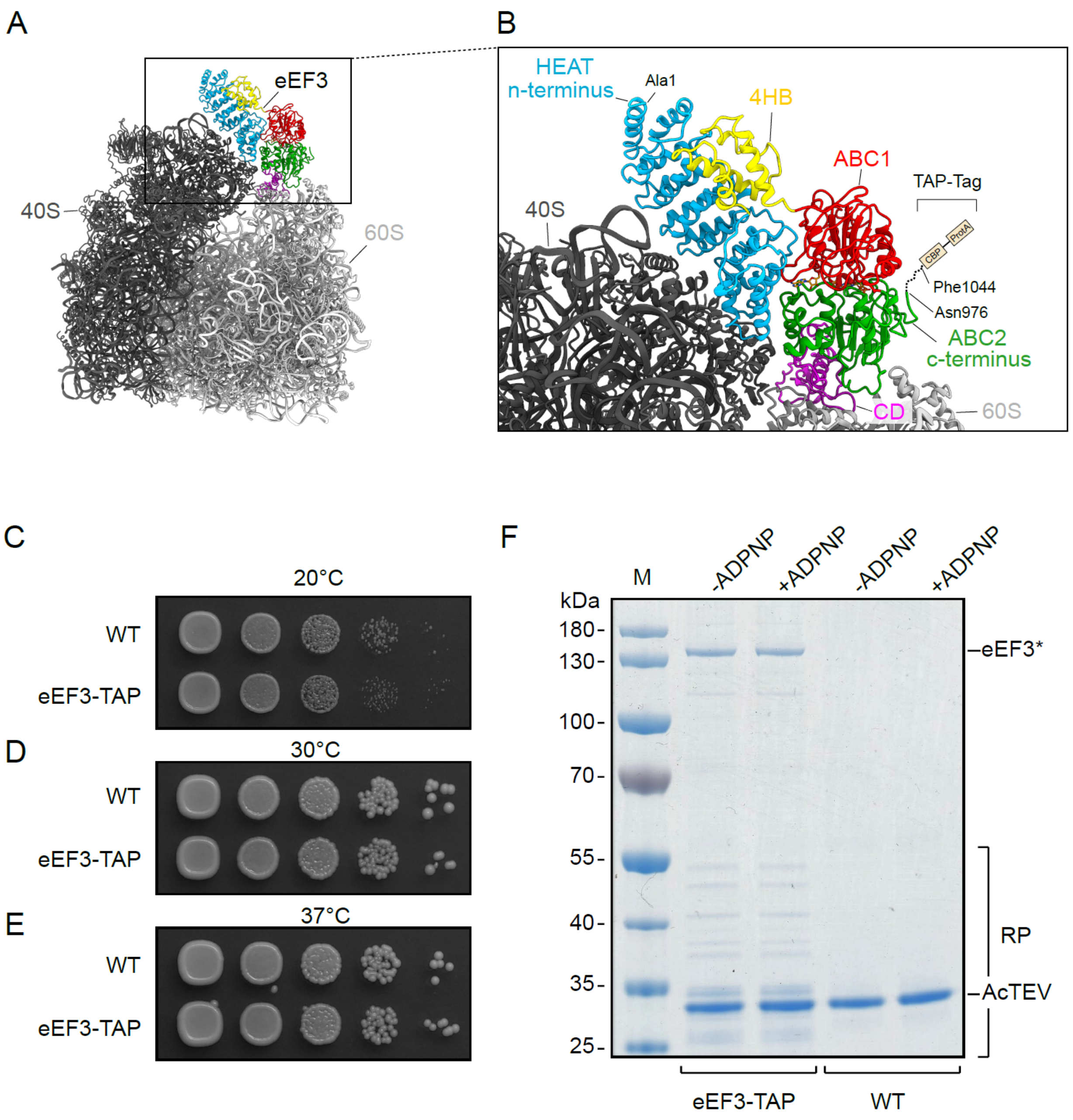

Appendix Figure S2. Analysis of eEF3-TAP tagged strains and purifications, related to Figure 4. (**A**) Overview and (**B**) Zoom of binding position of eEF3 on the 80S ribosome, highlighting in (**B**) the position of the C-terminal TAP-tag. (**C-E**) Growth of WT and eEF3-TAP tagged strain as a dilution series on agar plates incubated at (**C**) 20°C, (**D**) 30°C and (**E**) 37°C. (F) Coomassie stained SDS-PAGE of eluation fractions of tandem affinity purification using eEF3-TAP and WT strains in the absence and presence of ADPNP. M, molecular weight marker (kDa); AcTEV indicates position of TEV protease, eEF3*, position of eEF3 and RP, ribosomal proteins

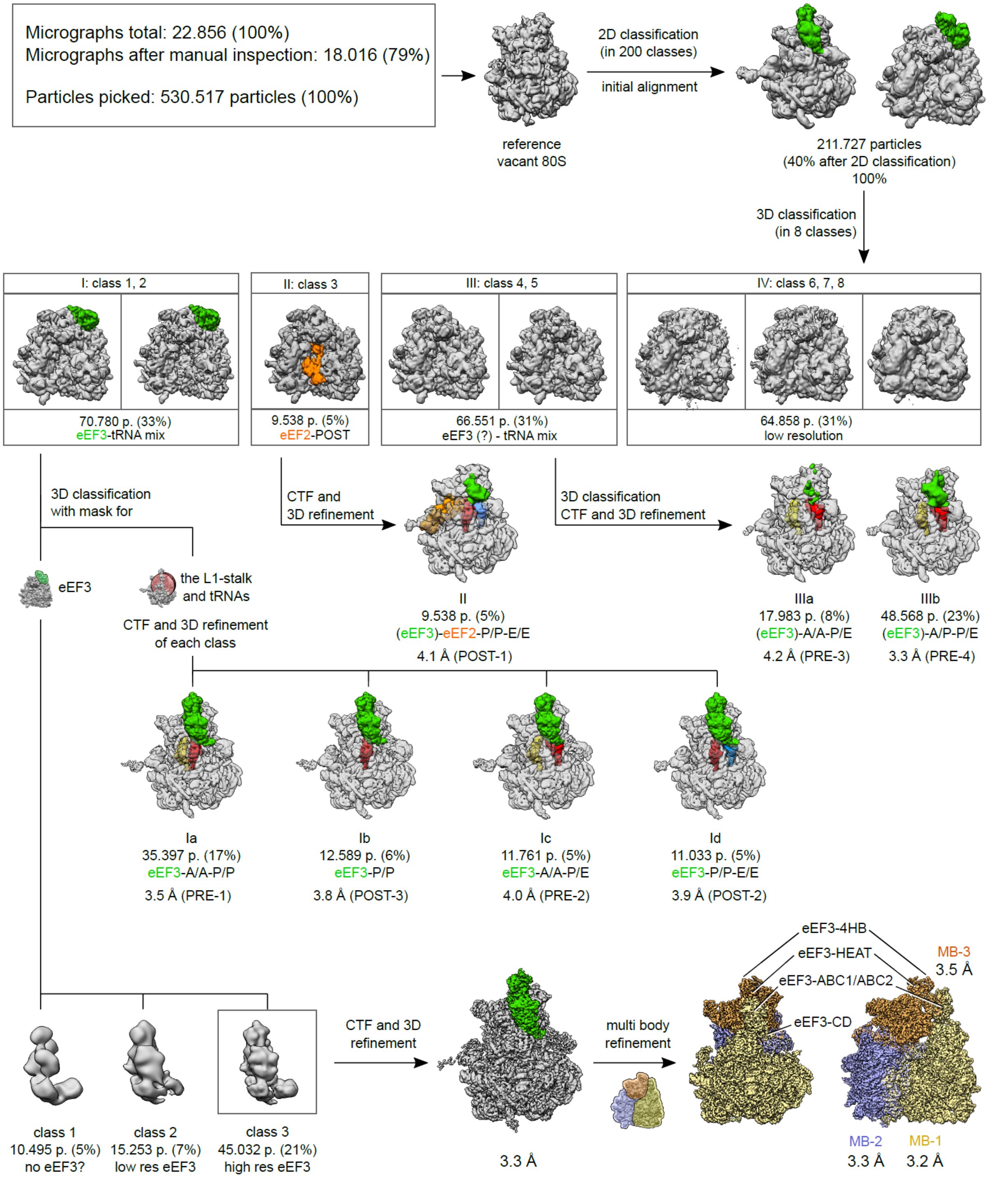

Appendix Figure S3. 3D classification of the *S. cerevisiae* eEF3-80S complex, related to Figure 4. Following 2D classification, 211,727 particles were initially aligned against a vacant *S. cerevisiae* 80S ribosome and subjected to 3D classification, sorting the particles into eight classes. The particles in class 1 and 2 bearing a stable eEF3-80S complex were joined in group I and subjected to two focused sortings using two different masks: the first encompassing the eEF3

ligand and the second covering the tRNAs, the L1-stalk and the eEF3-CD. The first classification allowed the sorting for a high resolution eEF3 bound volume (class 3, 21%, 45,032 particles), which was 3D and CTF refined resulting in a 3.3 Å final reconstitution. The final map was further multi body refined and provided a resolution of 3.2 Å for the LSU-eEF3 (ABC1/2, CD) (MB-1), 3.3 Å for the SSU body (MB-2) and 3.5 Å for the SSU head-eEF3 (HEAT, 4HB) (MB-3). The second sorting procedure enabled the classification of four EF3-bound non-rotated ribosomal classes with distinct tRNA occupancies (Ia-Id) with final resolutions denoted in the scheme. Class 3 (group II) showed a ribosomal species bearing eEF2, P/P- and E/E-tRNA and was finally refined to 4.1 Å. Class 4 and 5 (joined to group III) showed a rotated ribosome with mixed tRNA occupancy and after low pass filtering a disordered eEF3 ligand. This class was sorted further into two volumes (IIIa, IIIb) whereas each of them was 3D and CTF refined resulting in 4.2 Å and 3.8 Å resolution for the A/A- and P/E-tRNA occupied 80S (IIIa) and the A/P and P/E-tRNA bound ribsome (IIIb), respecively. Classes 6, 7, 8 (group IV) contained low resolution particles, which also showed a partial density for eEF3.

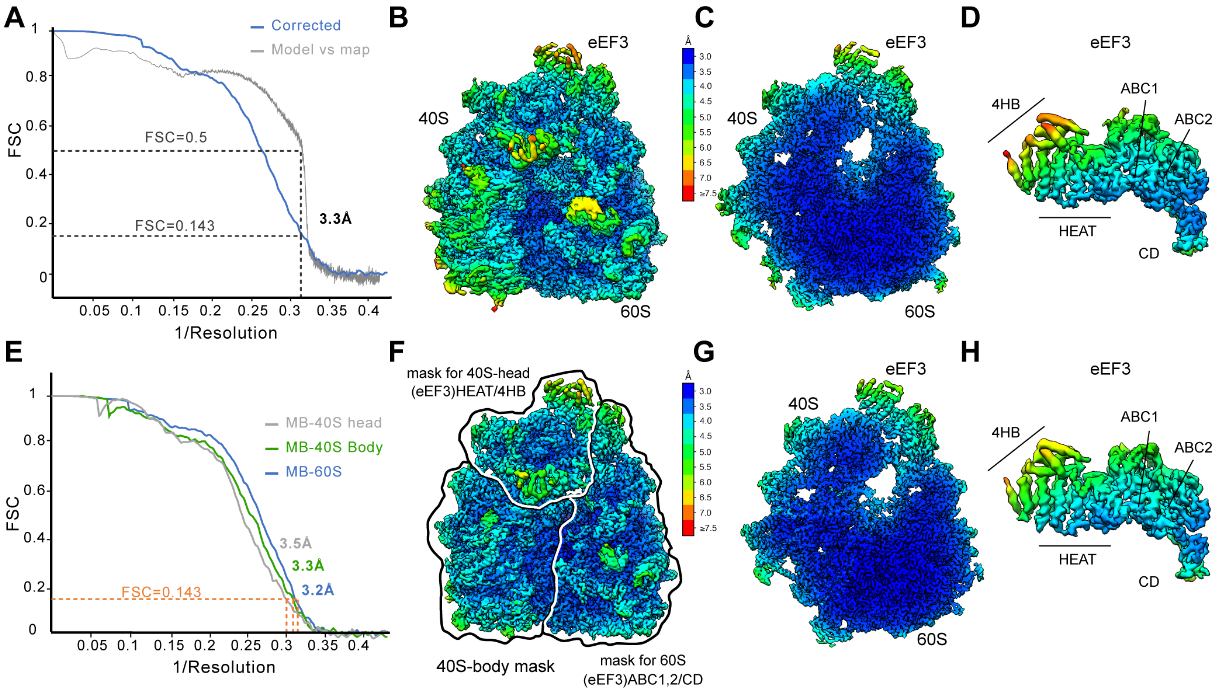

Appendix Figure S4. Overview of the final refined cryo-EM reconstruction of the eEF3-80S complex, related to Figure 4. (**A**) Fourier shell correlation (FSC) curve (blue curve) of the final refined cryo-EM map of the eEF3 ribosomal complex, indicating the average resolution of 3.3 Å, according to the gold-standard criterion (FSC=0.143). Overlay is the FSC curve calculated between the cryo-EM reconstruction and the final model (grey curve). (**B**) Cryo-EM map of the 3.3 Å EF3-80S complex filtered and colored according to local resolution and (**C**) its transverse section. (**D**) View of the isolated density for eEF3 from (**B**). (**E**) FSC curve of the three multibody (MB) refined cryo-EM map of the eEF3-80S complex, indicating the average resolution of 3.2 Å for MB-60S, 3.3 Å for MB-40S body and 3.5 Å for MB-40S head. (**F**) Cryo-EM map of the MB refined EF3-80S complex filtered and colored according to local resolution. The outlines are depicting the three masks, which were used for the MB refinement. (**G**) Transverse section of the volume shown in (**F**). (**H**) Isolated density for eEF3 from (**F**). The scale bar is valid for all showed resolution panels.

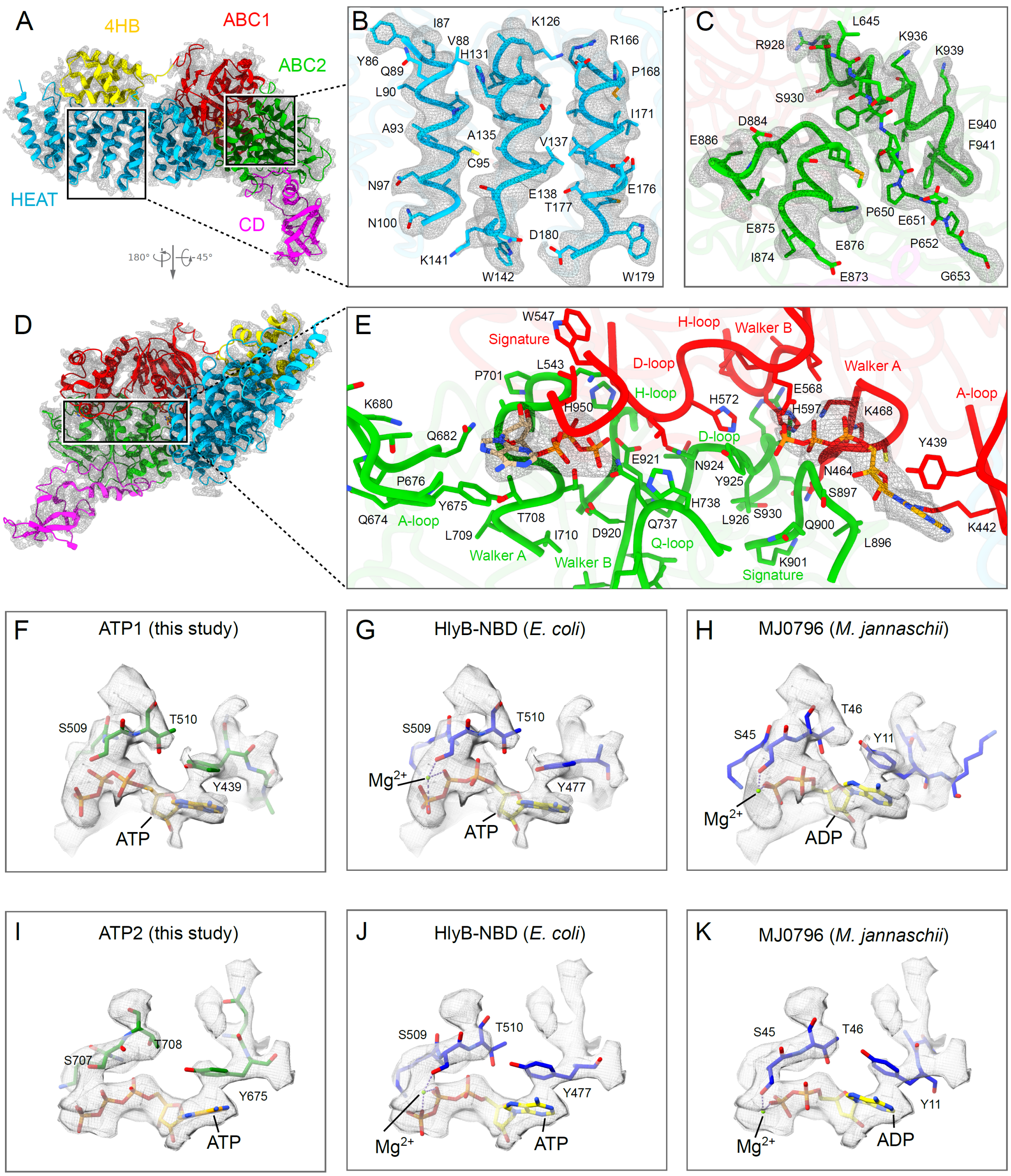

Appendix Figure S5. Model for *S. cerevisiae* eEF3 with bound ATP/ADPNP molecules, related to Figure 4. (**A**) Model of the 80S-bound eEF3 based on the multibody refined map (gray mesh) and colored by domain. HEAT (blue), 4HB (yellow), ABC1 (red), ABC2 (green) and CD (magenta). (**B-C**) Selected examples illustrating the quality of fit of the molecular model within (**B**) the HEAT repeat region and (**C**) the ABC2 domain to the unsegmented cryo-EM map (gray mesh). (**D**) eEF3-80S molecular model shown in (**A**), but in the orientation showing the

ATP-binding cassettes and (**E**) zoom of the two nucleotide-binding sites formed by ABC1 and ABC2. For the bound ATP/ADPNP molecules cryo-EM map densities of the multibody refined map (dark grey mesh) are illustrated. The residues, which show density are shown as sticks and labelled. (**F-K**) Isolated electron density (transparent grey) for nucleotide binding pocket of (**F-H**) ABC1 and (**I-K**) ABC2 modelled with (**F, I**) ATP in the eEF3 (green) structure, or rigid-body fitted with the crystal structures of (**G,J**) *E. coli* HlyB-NBD with ATP/Mg^{2+} (PDB ID: 1XEF) (Zaitseva, Jenewein, Jumpertz, Holland, & Schmitt, 2005) or (**H,K**) *M. jannaschii* MJ0796 ABC protein with ADP/Mg^{2+} (PDB ID: 1F3O) (Yuan et al., 2001).

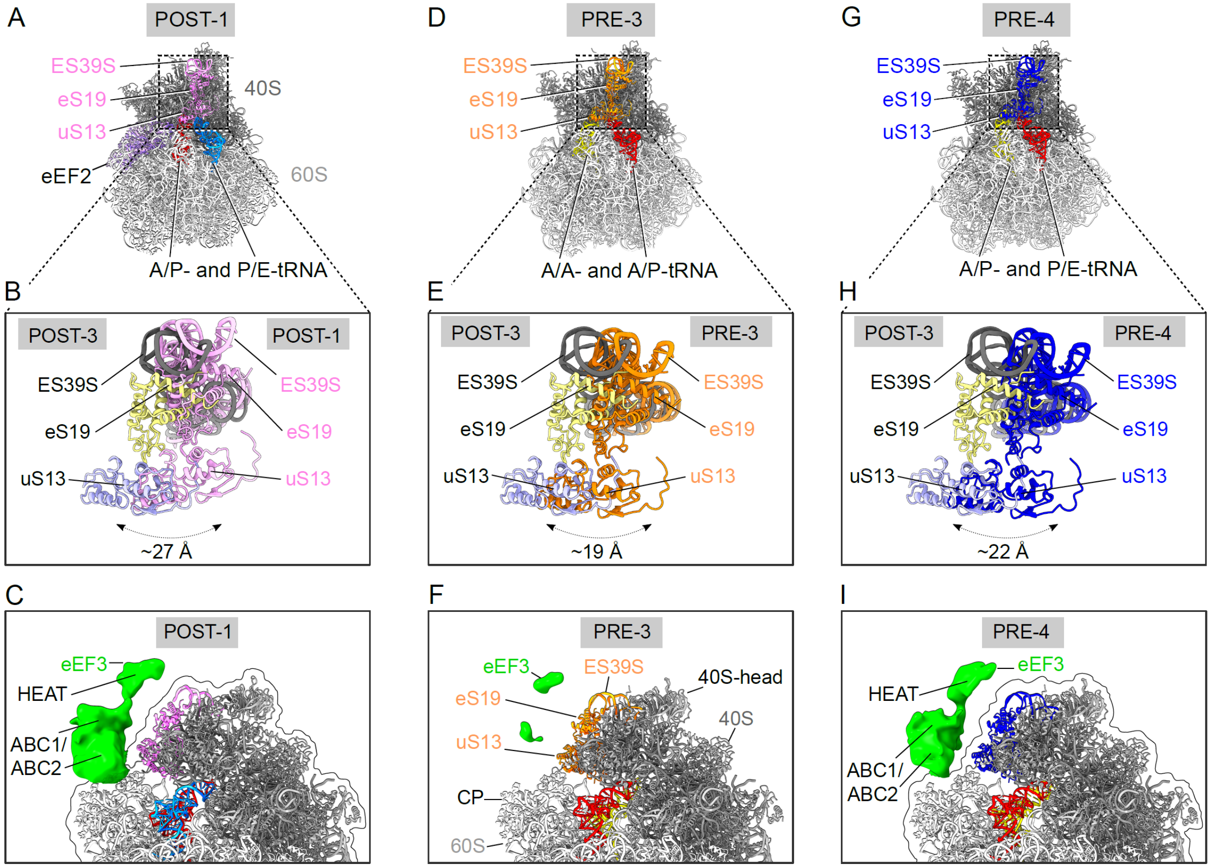

Appendix Figure S6. The rotated 80S bound to disordered eEF3, related to Figure 6. (**A**) Rotated 80S molecular model corresponding to the POST-1 state (disordered density for eEF3 is not shown). The ribosomal residues ES39S, eS19 and uS13 responsible for eEF3-HEAT binding are depicted in pink. (**B**) Zoomed view of ES39S, eS19 and uS13 highlighted in (**A**) overlaid with corresponding residues from the non-rotated POST-3 state. The arrows are showing the magnitude of the movement of these residues from a non-rotated POST-3 to a rotated POST-1 state in (**A**). (**C**) Segmented density for the disordered eEF3 from the POST-1 cryo-EM map highlighting the lack of association of the eEF3-HEAT repeat region with the moved ES39S, eS19 and uS13 resulting from the subunit rotation. (**D**) 80S model in rotated PRE-3 state depicting ES39S, eS19 and uS13 in orange and the (**E**) corresponding zoom of the displacement of the mentioned residues in the rotated ribosome. (**F**) Isolated density for the potential disordered ligand from the PRE-3 volume showing barely any density for eEF3. (**G**) PRE-4 ribosomal model with ES39S, eS19 and uS13 colored in blue as well as (**H**) the corresponding enlargement of these residues compared to the residues in the POST-3 state. (**I**) Segmented density of the eEF3 ligand from the rotated PRE-4 state. The isolated densities in (**C**), (**F**) and (**I**) were low pass filtered to 8 Å.

Appendix Table S1 Cryo-EM data collection, refinement and validation statistics

	eEF3-80S complex
**PDB ID**	7B7D
**EMDB ID**	EMD-12081
**Data collection and processing**	
Microscope	Titan Krios
Magnification	129,151
Voltage (kV)	300
Electron exposure (e^-/Å^2)	28
Defocus range (μm)	-0.8 to -2.5
Pixel size (Å)	1.084
Symmetry imposed	C1
Initial particle images (no.)	530,517
Final particle images (no.)	45,032
Map resolution (Å)	3.3
FSC threshold	0.143
Map resolution range (Å)	2.9-7.5
Detector	Falcon 2
Map sharpening *B* factor (Å^2)	-88,2563
**Refinement**	
Initial model used (PDB code)	6S47
CC_{Volume}	0.77
CC_{Mask}	0.78
CC_{Box}	0.75
Model composition	
Non-hydrogen atoms	211,145
Protein residues	12,111
RNA bases	5,430
R.m.s. deviations	
Bond lengths (Å)	0.010
Bond angles (°)	1.509
Validation	
MolProbity score	1.75
Clashscore	4.35
Poor rotamers (%)	0.99
Ramachandran plot	
Favored (%)	90.56
Allowed (%)	9.35
Disallowed (%)	0.08
Validation RNA	
Correct sugar pucker (%)	98.5
Good backbone conf. (%)	97.9

Supplemental References

Barthelme, D., Dinkelaker, S., Albers, S. V., Londei, P., Ermler, U., & Tampe, R. (2011). Ribosome recycling depends on a mechanistic link between the FeS cluster domain and a conformational switch of the twin-ATPase ABCE1. *Proc Natl Acad Sci U S A, 108*(8), 3228-3233. doi:10.1073/pnas.1015953108

Crowe-McAuliffe, C., Graf, M., Huter, P., Takada, H., Abdelshahid, M., Novacek, J., . . . Wilson, D. N. (2018). Structural basis for antibiotic resistance mediated by the Bacillus subtilis ABCF ATPase VmlR. *Proc Natl Acad Sci U S A, 115*(36), 8978-8983. doi:10.1073/pnas.1808535115

Nurenberg-Goloub, E., Kratzat, H., Heinemann, H., Heuer, A., Kotter, P., Berninghausen, O., . . . Beckmann, R. (2020). Molecular analysis of the ribosome recycling factor ABCE1 bound to the 30S post-splitting complex. *EMBO J, 39*(9), e103788. doi:10.15252/embj.2019103788

Webb, K. J., Al-Hadid, Q., Zurita-Lopez, C. I., Young, B. D., Lipson, R. S., & Clarke, S. G. (2011). The ribosomal l1 protuberance in yeast is methylated on a lysine residue catalyzed by a seven-beta-strand methyltransferase. *J Biol Chem, 286*(21), 18405-18413. doi:10.1074/jbc.M110.200410

Yuan, Y. R., Blecker, S., Martsinkevich, O., Millen, L., Thomas, P. J., & Hunt, J. F. (2001). The crystal structure of the MJ0796 ATP-binding cassette. Implications for the structural consequences of ATP hydrolysis in the active site of an ABC transporter. *J Biol Chem, 276*(34), 32313-32321. doi:10.1074/jbc.M100758200

Zaitseva, J., Jenewein, S., Jumpertz, T., Holland, I. B., & Schmitt, L. (2005). H662 is the linchpin of ATP hydrolysis in the nucleotide-binding domain of the ABC transporter HlyB. *EMBO J, 24*(11), 1901-1910. doi:10.1038/sj.emboj.7600657
