# Supplementary material for: Yeast translation elongation factor eEF3 promotes late stages of tRNA translocation
Source: EMBO J. 2021 Feb 8;40(6):e106449. doi: 10.15252/embj.2020106449 (PMC7957392; doi:10.15252/embj.2020106449)
Supplement: Supplementary file 2 — Expanded View Figures PDF [file EMBJ-40-e106449-s007.pdf]

Expanded View Figures

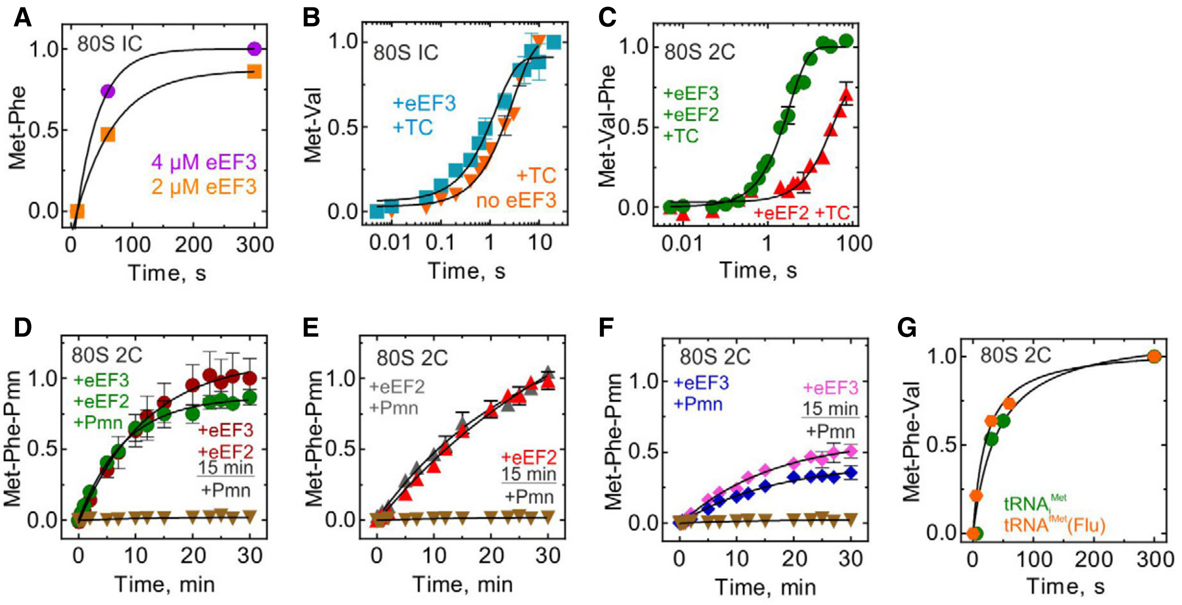

Figure EV1. Di- and tripeptide formation.

A Time courses of MetPhe formation at different eEF3 concentrations. Data are normalized to Met-Phe formation in the presence of 4 μM eEF3 with the maximum value in the dataset set to 1.

B Met-Val formation monitored upon rapidly mixing initiation complexes (80S IC; 1 μM) with ternary complexes eEF1A–GTP–[^{14}C]Val-tRNA^{Val} (0.2 μM) in the presence (cyan, 0.78 ± 0.1/s) or absence (orange, 0.34 ± 0.03/s) of eEF3 in a quench-flow apparatus, and the extent of peptide formation was analyzed by HPLC and radioactivity counting. Data are normalized to Met-Val formation in the presence of eEF3 with the maximum value in the dataset set to 1. Data presented as mean ± SEM of n = 3 biological replicates.

C Met-Val-Phe formation upon rapid mixing of 80S complexes carrying MetVal-tRNA^{Val} (80S 2C) with ternary complexes eEF1A–GTP–[^{14}C]Phe-tRNA^{Phe} in the presence of eEF2 and eEF3 (green, 0.3 ± 0.02/s), eEF2 (red, 0.03 ± 0.006/s). Data are normalized to Met-Val-Phe formation in the presence of eEF2 and eEF3 with the maximum value in the dataset set to 1. Data presented as mean ± SEM of n = 3 biological replicates.

D–F Comparison of time courses of 80S 2C reaction with Pmn. 80S 2C complexes with MetPhe-tRNA^{Phe} in the presence of eEF2 and eEF3 (D), eEF2 (E) or eEF3 (F), or in the absence of eEF2 and eEF3 (brown triangles), with Pmn in a quench-flow apparatus. As indicated, the reaction was started either by mixing all components, or by addition of Pmn to a mixture of 80S 2C with the factors preincubated for 15 min. The extent of MetPhe-Pmn formation was analyzed by HPLC and radioactivity counting. Data are normalized to Met-Phe-Pmn formation in the presence of eEF2 and eEF3 (D), eEF2, (E) or eEF3 (F) with the maximum value in the dataset set to 1. Data presented as mean ± SEM of n = 3. For comparison, data from Fig 2B are plotted.

G Met-Phe-Val formation upon rapid mixing of 80S complexes carrying either [^{3}H]Met-tRNA$_i^{Met}$ (green) or [^{3}H]Met-tRNA^{fMet}(flu) (orange) MetPhe-tRNA^{Phe} (80S 2C) with ternary complexes eEF1A–GTP–[^{14}C]Val-tRNA^{Val} in the presence of eEF2 and eEF3. Data are normalized to Met-Phe-Val formation in the presence of non-labeled initiator tRNA (green) with the maximum value in the dataset set to 1.

Source data are available online for this figure.

© 2021 The Authors

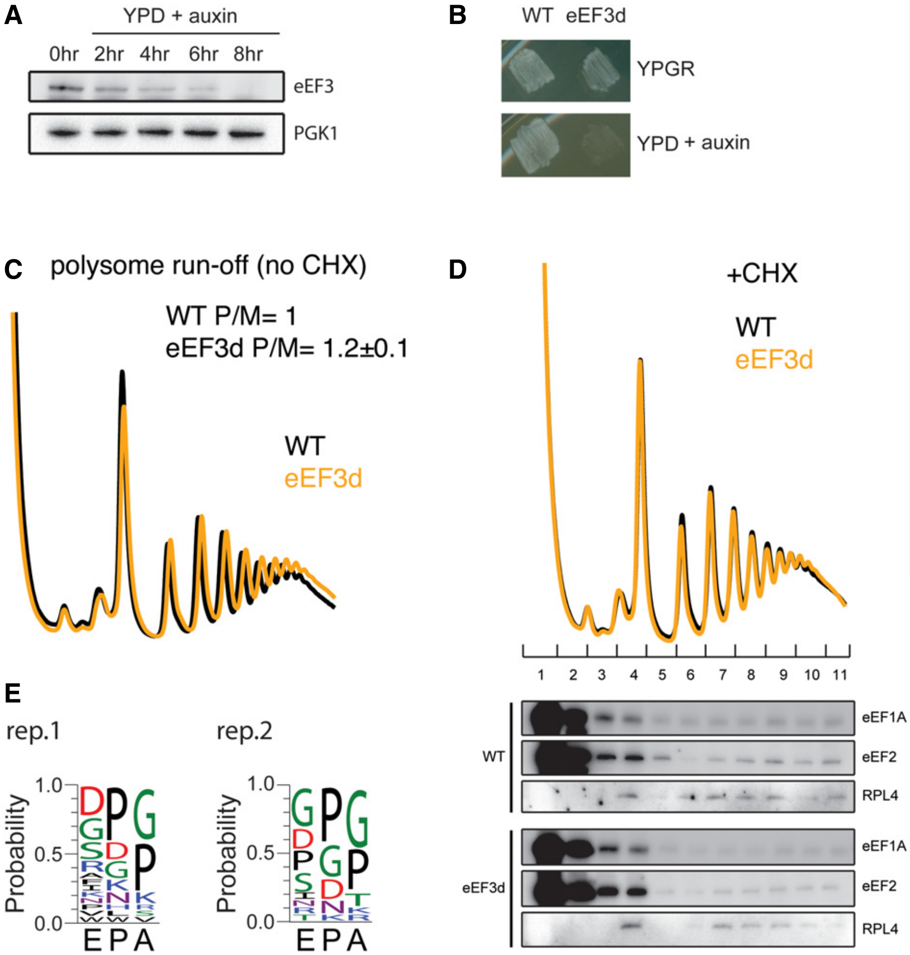

Figure EV2. Analysis of *in vivo* ribosome functional states by ribosome profiling.

A Immunoblot of eEF3 depletion over time. Same amount of cells were harvested at indicated time points, lysed, and subjected to immunoblotting using antibodies against eEF3 or PGK1.

B Growth of WT and eEF3d cells on YPGR and YPD + auxin plates. Plates were incubated at 30°C for 1 day.

C Representative run-off polysome profiles for WT and eEF3d strains. Polysome-to-monosome ratios (P/M) are normalized to WT ratios. Data are presented as mean ± SD, $n = 2$.

D Polysome profiles from WT or eEF3d cells with CHX added during cell lysis to stop translation (top). Fractions were analyzed by immunoblotting using antibodies against eEF1A, eEF2, or RPL4 (bottom).

E De-enriched peptide motifs associated with ribosome pausing at the E, P, and A sites in the absence of eEF3. Peptide motif logos from two biological replicates are shown.

 © 2021 The Authors

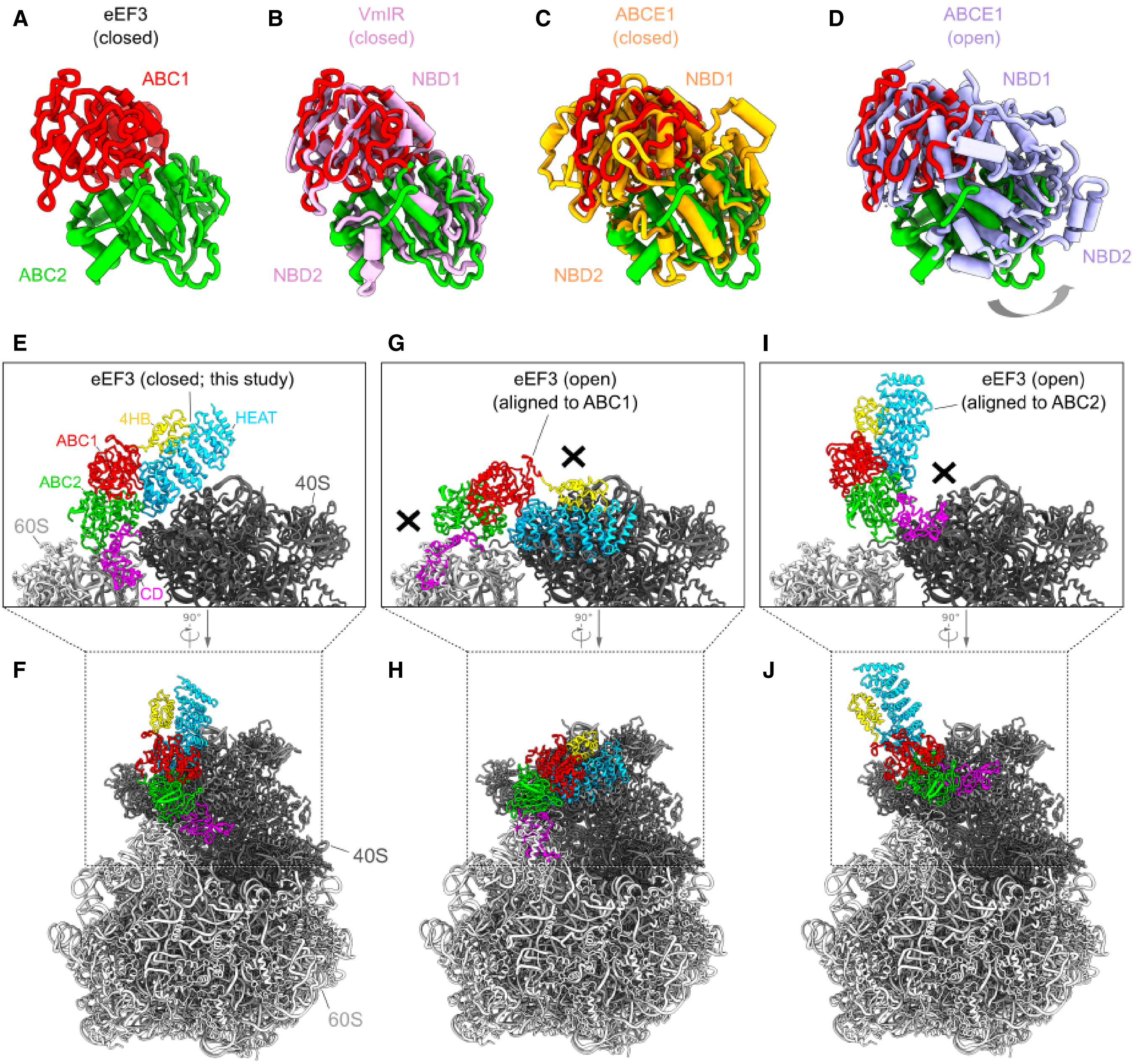

Figure EV3. The closed and open conformations of eEF3.
The closed and open conformations of eEF3

A The ABC1 (red) and ABC2 (green) domain of eEF3 in the eEF3-80S complex.

B–D Conformation of the eEF3 NBDs with respect to other ABC proteins. Alignment (based on ABC1) of the eEF3-ABCs with (B) the closed conformation of the NBDs of the *B. subtilis* ABCF ATPase VmlR (pink, PDB: 6HA8) (Crowe-McAuliffe *et al*, 2018), (C) the closed conformation of the archaeal ABCE1-30S post-splitting complex (orange, PDB: 6TMF) (Nurenberg-Goloub *et al*, 2020), and (D) the *E. coli* ABCE1 protein observed in the open conformation (violet, PDB ID: 3OZX) (Barthelme *et al*, 2011).

E, F The eEF3 model in a closed conformation colored due to its different domain organization.

G–J Incompatibility of eEF3 to the 80S ribosome in a potential opened conformation. The eEF3 model was aligned to (G, H) ABC1 or (I, J) ABC2 of the ABCE1 protein in an opened conformation (PDB ID: 3OZX) (Barthelme *et al*, 2011).

© 2021 The Authors

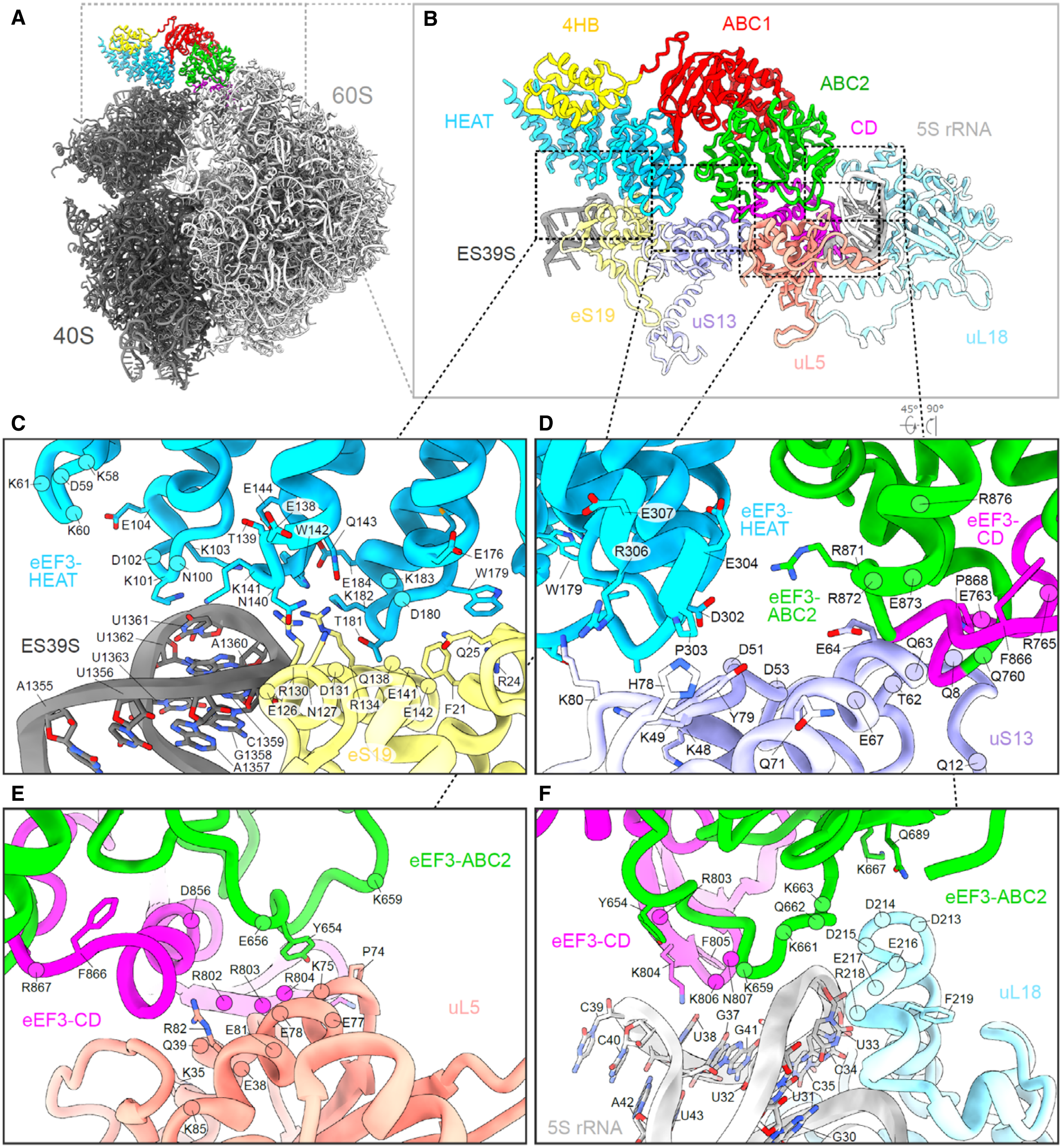

Figure EV4. Interactions of eEF3 with the 80S ribosome.

A, B (A) Overview of the eEF3-80S molecular model with (B) zoom on the eEF3 model (colored by domain) interacting with ribosomal components.
C Interactions of the eEF3-HEAT (blue) with ES39S of the 18S rRNA (dark gray) and eS19 (pale yellow) of the SSU.
D The 40S protein uS13 (violet) forms bridging contacts with the HEAT (blue), ABC2 (green), and CD (magenta) domains of eEF3.
E, F The eEF3-CD and eEF3-ABC2 interact with (E) the LSU protein uL5 (coral) and (F) the 5S rRNA (light gray) and uL18 (pale blue).

 © 2021 The Authors

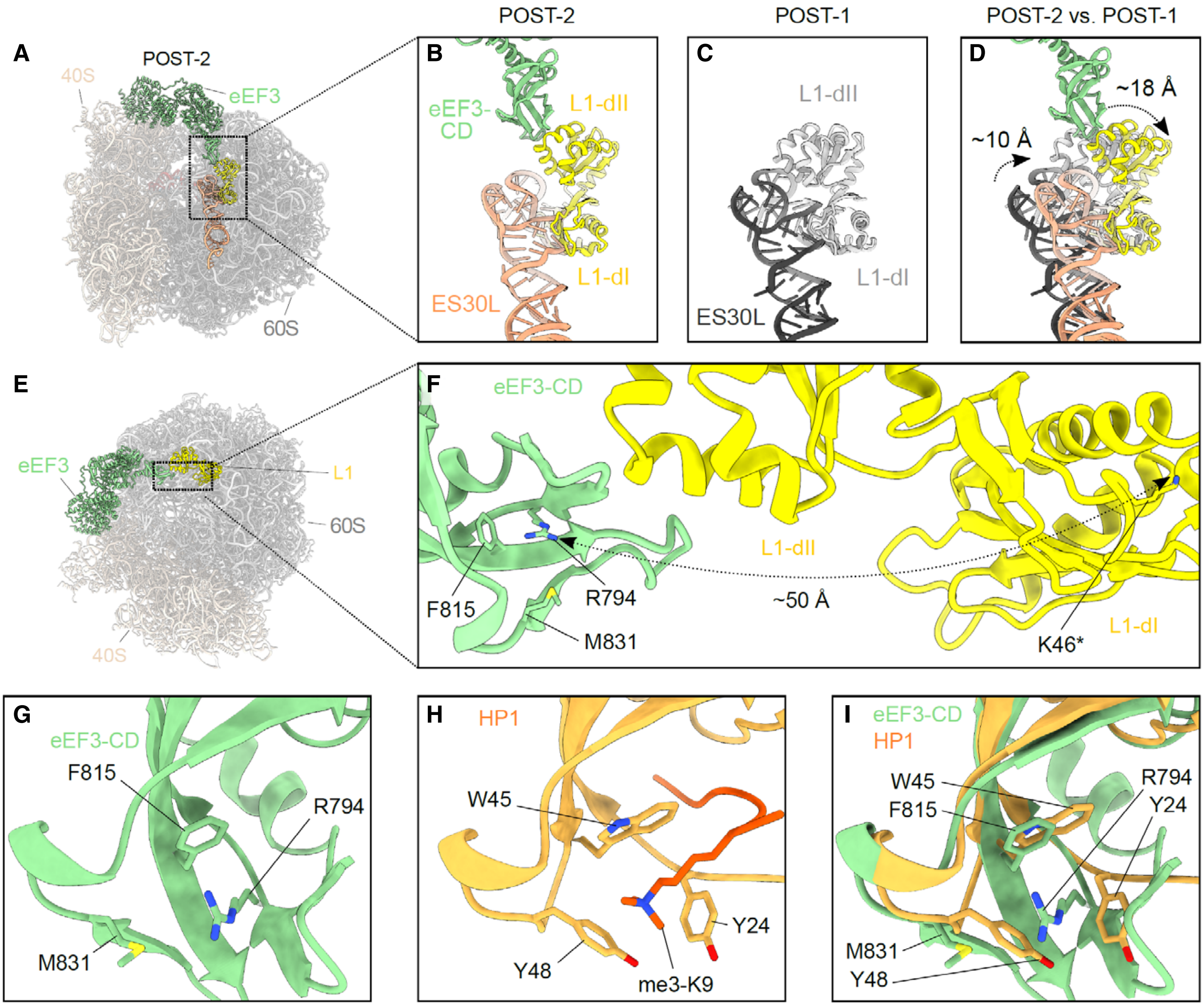

Figure EV5. The hydrophobic pocket of the eEF3 CD.

A, B (A) Overview of the POST-2 eEF3-80S molecular model (B) highlighting the position of eEF3-CD (green) relative to L1 protein (yellow) and ES30L (orange).

C, D (C) Same as (B) but for POST-1, and (D) comparison of (B) and (C) depicting the magnitude of the L1-stalk movement from the POST-2 state (L1-stalk'int') to the POST-1 state (L1-stalk'in'). L1-POST1 (light gray), ES30L-POST1 (dark gray).

E, F (E) eEF3-80S molecular model highlighting eEF3 (pale green) and the L1 protein (yellow) and its (F) zoom showing the magnitude of the distance between the eEF3-CD hydrophobic pocket (based on the alignment with HP1 shown in (I)) and the K46 of the L1-dI. K46* labels the lysine, which is getting methylated by the Seven-β-strand methyltransferase (Webb *et al*, 2011).

G, H (G) The eEF3-CD hydrophobic pocket based on the alignment with (H) the *D. melanogaster* HP1-CD.

I Overlay of the eEF3 and HP1 CD based on their sequence.

© 2021 The Authors
